# Behavioral recommendations following gynecological surgery

**DOI:** 10.1007/s00508-025-02548-0

**Published:** 2025-05-30

**Authors:** Felix Hofbauer, Laura Strobel, Christian Marth, Andreas Widschwendter

**Affiliations:** Department of Gynaecology and Obstetrics, Medical University of Innsbruck, Anichstraße 35, 6020 Innsbruck, Austria

**Keywords:** Postoperative care, Clinical practice variation, Recovery time, Rehabilitation, Patient education

## Abstract

**Background:**

Postoperative care is critical for recovery after gynecological procedures. Yet, the absence of consistent guidelines often leaves patients uncertain, potentially hindering healing. This study aimed to assess behavioral recommendations provided by Austrian gynecologists and hospitals to inform the development of standardised postoperative guidance.

**Patients and methodology:**

Between December 2023 and April 2024, questionnaires were sent to 118 gynecologists and 79 hospitals, covering postoperative advice for hysteroscopy, large loop excision of the transformation zone (LLETZ) conisation, tension-free vaginal tape (TVT), laparoscopy, hysterectomy, and caesarean section. Topics included duration of sick leave, follow-up, bathing, tampon use, sexual activity, sports, and lifting restrictions.

**Results:**

Responses were received from 54 gynecologists (46%) and 49 clinics (62%). Clinics tended to recommend more restrictive measures than specialists, particularly for LLETZ, TVT, and laparoscopy, while advice aligned for hysterectomy and caesarean section. Notably, for LLETZ conisation, specialists advised 2–4 weeks of rest, whereas clinics recommended 1–2 weeks (*p* < 0.05). For hysterectomy and caesarean section, both groups advised 4–6 weeks of recovery, with restrictions on weight lifting, sexual activity, and sports. Variability was also seen in the basis for recommendations: 41% of specialists relied on personal experience, while 41% of clinics cited current evidence.

**Conclusion:**

This study highlights significant heterogeneity in postoperative advice across Austria and underscores the need for evidence-based, standardised guidelines to improve the quality and consistency of care in gynecology.

## Introduction

The postoperative management of gynecological patients is crucial for a rapid and complication-free recovery. Despite advances in postoperative care, with reduced hospitalization times and an increased range of surgical procedures, there is currently a lack of standardized guidelines for postoperative behavioral recommendations in gynecology; however, studies by Bisgaard et al. [1] and Ottesen et al. [2] have demonstrated that the implementation of standardized postoperative counselling significantly reduces the duration of sick leave and consequently leads to substantial cost savings.

The development of a standardized postoperative management protocol is complicated by the wide variability of gynecological procedures and the multitude of factors that need to be considered in gynecological aftercare. These include the duration of sick leave, physical rest, and the timing of follow-up appointments as well as restrictions on sexual activity, tampon use, sauna visits and swimming. Furthermore, the frequent scheduling of outpatient surgery results in limited patient contact, which in turn complicates patient education [3].

The current state of research on postoperative behavioral measures following gynecological surgery is inadequate. In contrast to the current state of guidelines in the field, which only provide limited information on postoperative management, the Royal College of Obstetricians and Gynaecologists developed a precise recommendation catalogue for specific activities in 2012, in collaboration with the Department of Work and Pensions [4]. The lack of evidence-based recommendations has resulted in persistent reliance on conventional practices that are largely based on the personal experiences of medical professionals. Güsgen et al. [5] demonstrated in a nationwide survey in Germany that 78% of respondents based their recommendations on personal experience, while only 11% relied on current research evidence.

The two most extensively studied factors in postoperative behavioral recommendations are physical activity and the duration of sick leave, both of which show significant variability. A systematic review by Moller et al. [6] on postoperative behavioral recommendations after hysterectomy found a wide range of recommendations, ranging from 0 to 12 weeks. In a similar manner, Naidu et al. [4] identified substantial variations in the duration of convalescence periods and timeframes of return to work as well as significant variations in recommendations for resuming sports and sexual activities.

Despite the fact that the enhanced recovery after surgery (ERAS) concept has been in existence for some time, there is little information regarding postoperative recommendations [7]. The ERAS initiative, which focuses on perioperative care, is becoming increasingly more important in the field of gynecology; however, of the 10 postoperative components described in the ERAS framework, only 1 relates to aftercare, stating that patients should receive “specific instructions regarding recovery time and emergency contacts” [8]. There is no evidence for the resumption of work, physical activity, or sexual activity.

The objective of the present study is to optimize and standardize the postoperative care of gynecological patients, thereby establishing a foundation for evidence-based guidelines. The implementation of these findings is expected to enhance the consistency of recommendations among clinics and specialists.

## Methods

A nationwide survey was conducted in Austria between December 2023 and April 2024. The survey was administered via the online platform “SurveyMonkey”, and responses were collected at two time points. The questionnaire was distributed via the mailing list of the Austrian Society for Gynecology and Obstetrics (OEGGG) and the Professional Association for Obstetrics and Gynecology. A total of 118 gynecologists in private practice in Tyrol and 79 hospitals with departments of gynecology and obstetrics across Austria were contacted. The questionnaire was addressed to the management board of the respective clinic and was to be completed by the board itself or by a senior consultant. It was imperative that each questionnaire was completed by only one person. It is important to note that the questionnaire explicitly excluded considerations of pre-existing conditions or professional limitations.

In the initial phase of the study, respondents were invited to specify the foundation of their recommendations, with the following options being provided: current research evidence, expert opinion, personal experience, institutional guidelines (termed house opinion), or the option of stating that none of the aforementioned applied. Subsequently, behavioral recommendations were requested for hysteroscopy, large loop excision of the transformation zone (LLETZ) conization, tension-free vaginal tape (TVT), laparoscopy, hysterectomy and cesarean section. For each procedure the following parameters were evaluated: recommended sick leave duration, physical rest, timing of follow-up specialist appointments, restrictions on full baths, tampon usage, sexual intercourse, sporting activities (in weeks), and limitations on lifting heavy objects (in kg). Each question provided four possible responses. The data were analyzed using IBM SPSS Statistics Version 26 (IBM Corp. Released 2020. IBM SPSS Statistics for Windows, Version 26.0. Armonk, NY, USA: IBM Corp). The mode was used to evaluate the most frequent responses. The χ^2^-test was employed to compare two or more groups, with a *p*-value of < 0.05 considered statistically significant.

## Results

The response rate for clinics was 62%, with a total of 49 included. The response rate among specialists was 46%, with 54 questionnaires out of a total of 118 returned. The values that occur most frequently in the sample (in %) of all questions from the total collective (clinics and specialists) are displayed in Table 1. A comparison of the responses from both groups reveals that clinics tend to give more limited recommendations and restrictions compared to specialists. This discrepancy is most pronounced in the cases of LLETZ conization, TVT and laparoscopy. In contrast, the recommendations of specialists and clinics are congruent for hysterectomy and cesarean section. The individual recommendations of the clinics and specialists are shown separately in Tables 2 and 3.

**Table 1:** Modal values of overall collective (clinics *n* = 49 and medical specialists *n* = 54)

	Hysteroscopy	LLETZ	TVT	Laparoscopy	Hysterectomy	Cesarean section
Duration of sick leave	Up to 1 week (75%)	1–2 weeks (49%)	1–2 weeks (43%)	1–2 weeks (53%)	3–6 weeks (62%)	3–6 weeks (47%)
Lifting and carrying	No restriction (56%)	5–10 kg (32%)	5–10 kg (40%)	5–10 kg (44%)	5–10 kg (43%)	5–10 kg (45%)
Specialist check-up	4–6 weeks (63%)	4–6 weeks (60%)	4–6 weeks (55%)	4–6 weeks (65%)	4–6 weeks (61%)	4–6 weeks (52%)
No full baths	1–2 weeks (41%)	2–4 weeks (55%)	2–4 weeks (39%)	1–2 weeks (42%)	4–6 weeks (63%)	4–6 weeks (50%)
No tampons	1–2 weeks (34%)	2–4 weeks (33%)	1–2 weeks (25%)	No restriction (50%)	4–6 weeks (47%)	4–6 weeks (45%)
No sexual intercourse	1–2 weeks (39%)	2–4 weeks (45%)	2–4 weeks (57%)	1–2 weeks (34%)	4–6 weeks (56%)	4–6 weeks (48%)
No physical activity	Up to 1 week (43%)	1–2 weeks (44%)	2–4 weeks (60%)	2–4 weeks (38%)	4–6 weeks (53%)	4–6 weeks (42%)

Data presented as modal values in %

*LLETZ* large loop excision of the transformation zone, *TVT* tension-free vaginal tape

**Table 2:** Modal values of clinics (*n* = 49)

	Hysteroscopy	LLETZ	TVT	Laparoscopy	Hysterectomy	Cesarean section
Duration of sick leave	Until 1 week (80%)	Until 1 week (45%)	Until 1 week (37%)	1–2 weeks (41%)	3–6 weeks (61%)	3–6 weeks (43%)
Lifting and carrying	No restriction (67%)	No restriction (45%)	1–5 kg = 5–10 kg (29%)	No restriction (37%)	5–10 kg (39%)	5–10 kg (39%)
Specialist check-up	4–6 weeks (61%)	4–6 weeks (57%)	4–6 weeks (49%)	4–6 weeks (61%)	4–6 weeks (61%)	4–6 weeks (55%)
No full baths	Until 1 week = 1–2 weeks (37%)	2–4 weeks (49%)	1–2 weeks (39%)	1–2 weeks (41%)	4–6 weeks (51%)	4–6 weeks (43%)
No tampons	1–2 weeks (33%)	1–2 weeks = 2–4 weeks (29%)	1–2 weeks (29%)	No restriction (57%)	4–6 weeks (37%)	4–6 weeks (37%)
No sexual intercourse	1–2 weeks (35%)	2–4 weeks (41%)	2–4 weeks (55%)	1–2 weeks (31%)	4–6 weeks (49%)	4–6 weeks (41%)
No physical activity	Until 1 week (35%)	1–2 weeks (43%)	2–4 weeks (51%)	1–2 weeks = 2–4 weeks (31%)	4–6 weeks (43%)	4–6 weeks (39%)

Data presented as modal values in %

*LLETZ* large loop excision of the transformation zone, *TVT* tension-free vaginal tape

**Table 3:** Modal values of medical specialists (*n* = 54)

	Hysteroscopy	LLETZ	TVT	Laparoscopy	Hysterectomy	Cesarean section
Duration of sick leave	Until 1 week (70%)	1–2 weeks (59%)	1–2 weeks (50%)	1–2 weeks (65%)	3–6 weeks (61%)	3–6 weeks (50%)
Lifting and carrying	No restriction (46%)	5–10 kg (44%)	5–10 kg (52%)	5–10 kg (54%)	5–10 kg (48%)	5–10 kg (50%)
Specialist check-up	4–6 weeks (65%)	4–6 weeks (63%)	4–6 weeks (61%)	4–6 weeks (70%)	4–6 weeks (61%)	4–6 weeks (61%)
No full baths	1–2 weeks (44%)	2–4 weeks (61%)	2–4 weeks (46%)	1–2 weeks (43%)	4–6 weeks (74%)	4–6 weeks (56%)
No tampons	1–2 weeks (35%)	2–4 weeks (39%)	2–4 weeks (39%)	No restriction (41%)	4–6 weeks (57%)	4–6 weeks (52%)
No sexual intercourse	1–2 weeks (43%)	2–4 weeks (48%)	2–4 weeks (59%)	1–2 weeks (37%)	4–6 weeks (63%)	4–6 weeks (54%)
No physical activity	Until 1 week (50%)	2–4 weeks (48%)	2–4 weeks (69%)	2–4 weeks (44%)	4–6 weeks (61%)	4–6 weeks (44%)

Data presented as modal values in %

*LLETZ* large loop excision of the transformation zone, *TVT* tension-free vaginal tape

### Hysteroscopy

The majority of medical specialists recommended a sick leave duration of up to 1 week (70%), whereas 30% advised a period of 1–2 weeks. In comparison, 80% of clinics recommended a sick leave of no more than 1 week. With respect to restrictions on heavy load lifting, 46% of specialists imposed no limitations, while 20% advised a maximum load of 5–10 kg. Among clinics, 67% imposed no restrictions, and only 10% recommended limiting lifting to 1–5 kg. In terms of recommendations concerning swimming and full baths, 44% of specialists advised a restriction period of 1–2 weeks, 26% recommended 1 week, and 28% suggested a duration of 2–4 weeks. Clinics tended to adopt more conservative guidance, with 37% each advising restrictions for up to 1 week and 1–2 weeks. With respect to tampon use, 33% of specialists recommended restrictions for up to 1 week, 35% for 1–2 weeks, and 24% for 2–4 weeks. A comparable distribution was observed among clinics, with 27% advising up to 1 week, 33% 1–2 weeks, and 20% 2–4 weeks. Concerning sexual activity, 43% of specialists recommended abstinence for 1–2 weeks, 32% for up to 1 week, and 24% for 2–4 weeks. A similar pattern was observed in the clinics, with 35% recommending 1–2 weeks, 29% up to 1 week and 22% 2–4 weeks. Half of the specialists (50%) recommended abstaining from sports for up to 1 week, while the remaining respondents suggested either 1–2 weeks restriction or no restriction. Among clinics, 35% advised a limitation of 1 week, whereas 31% placed no restriction on resuming sports activities. In most cases, both specialists and clinics recommend a follow-up consultation approximately 4–6 weeks postoperatively. A statistically significant discrepancy was observed between the recommendations of clinics and specialists with respect to the recommendation against bathing postsurgery (clinics: 37% 1–2 weeks; specialists: 44% 1–2 weeks, *p* = 0.047) and the utilization of tampons (clinics: 33% 1–2 weeks; specialists: 35% 1–2 weeks, *p* = 0.037).

### LLETZ conization

Among medical specialists, 19% recommended a sick leave of up to 1 week, 59% advised 1–2 weeks, and 19% suggested 2–4 weeks. Conversely, 45% of clinics recommended a duration of up to 1 week, and 37% suggested 1–2 weeks. Regarding restrictions on lifting heavy loads, 24% of specialists advised a limit of 1–5 kg, 44% recommended 5–10 kg, 17% permitted 10–15 kg, and 15% imposed no restrictions. Among clinics, 18% advised limits of 1–5 kg or 5–10 kg, while 45% imposed no restrictions. The majority of both specialists (63%) and clinics (57%) recommended a subsequent consultation within a period of 4–6 weeks. Restrictions on swimming and full baths were advised for 1–2 weeks by 35% of specialists and for 2–4 weeks by 61%. Clinics offered similar guidance, with 31% recommending 1–2 weeks and 49% suggesting 2–4 weeks. Regarding tampon use, 28% of specialists recommended restrictions of up to 1 week, 33% 1–2 weeks, and 39% 2–4 weeks. In clinics, 27% recommended up to 1 week, while 29% each advised 1–2 weeks and 2–4 weeks. For sexual activity, 24% of specialists advised abstinence for up to 1 week, 28% for 1–2 weeks, and 48% for 2–4 weeks. Clinic recommendations were similar, with 14%, 33%, and 41%, respectively. With respect to the resumption of sporting activities, 44% of specialists recommended a period of abstinence ranging from 1–2 weeks, while 48% recommended a duration of 2–4 weeks. Among clinics, 43% advised 1–2 weeks restriction, and 29% suggested 2–4 weeks. A significant discrepancy was observed between the recommendations of clinics and specialists concerning sick leave duration (clinics: 45% up to one week; specialists: 59% 1–2 weeks, *p* = 0.002). There was also a discrepancy in recommendations for sports restriction (clinics: 43% 1–2 weeks; specialists 48% 2–4 weeks, *p* = 0.001).

### Tension-free vaginal tape (TVT)

Among medical specialists, 17% recommended a sick leave of up to 1 week, 50% advised 1–2 weeks and 32% suggested 2–4 weeks. In comparison, 37% of clinics advised a sick leave of up to 1 week, and 35% recommended 1–2 weeks. Regarding lifting restrictions, 26% of specialists recommended limiting to 1–5 kg, 52% to 5–10 kg and 17% to 10–15 kg. Among clinics, 29% each advised restrictions of 1–5 kg and 5–10 kg, respectively, while 18% placed no restrictions. A follow-up consultation within a period of 4–6 weeks was recommended by 61% of medical experts and 49% of clinics. Restrictions on swimming and bathing were suggested for 1–2 weeks by 37% of specialists and for 2–4 weeks by 46% of clinics. Clinics exhibited slightly less restrictive guidance, with 39% recommending 1–2 weeks and 31% recommending 2–4 weeks. For tampon use, 30% of specialists advised 1–2 weeks of restriction, and 39% recommended 2–4 weeks. Clinics reported 29% for 1–2 weeks, 25% for 2–4 weeks, and 22% imposed no restrictions. Concerning sexual activity, 30% of specialists recommended abstinence for 1–2 weeks and 59% for 2–4 weeks. The recommendations made by clinics were found to be aligned with these figures, with 20% suggesting 1–2 weeks and 55% suggesting 2–4 weeks. Sports restrictions were advised by 22% of specialists for 1–2 weeks and 69% for 2–4 weeks. Among clinics, 16% recommended 1–2 weeks restriction, and 51% recommended 2–4 weeks, while 10% placed no restrictions or advised abstinence for up to 1 week. The recommendations of specialists and clinics diverged significantly in terms of the duration of absence from work (45% up to 1 week for clinics; 50% 1–2 weeks for specialists, *p* = 0.013).

### Laparoscopy

Among medical specialists, 65% recommended a sick leave of 1–2 weeks, and 24% suggested 2–4 weeks. In contrast, clinics advised up to 1 week in 20% of cases, 1–2 weeks in 41%, and 2–4 weeks in 25%. For lifting restrictions, 54% of specialists recommended a limit of 5–10 kg, while 13% advised 10–15 kg or placed no restrictions. Among clinics, 33% suggested limiting lifting to 5–10 kg, while 37% advised no restrictions. A postoperative consultation within 4–6 weeks was recommended by 70% of specialists and 61% of clinics. Restrictions on swimming and full baths were advised by 43% of specialists for 1–2 weeks and 37% for 2–4 weeks. Clinics recommended 1–2 weeks in 41% of cases and 2–4 weeks in 20%. In relation to the use of tampons, 24% of specialists recommended a restriction period of 1–2 weeks, while 41% did not impose any restrictions. Clinics similarly placed no restrictions in 57% of cases. For sexual activity, 37% of specialists recommended abstinence for 1–2 weeks and 30% for 2–4 weeks, while 17% placed no restrictions. Clinic recommendations were similar, with 31% advising 1–2 weeks, 22% 2–4 weeks, and 27% imposing no restriction. Regarding sports, 39% of specialists recommended 1–2 weeks restriction and 44% advised 2–4 weeks. Among clinics, 31% recommended both 1–2 and 2–4 weeks of restriction, while 18% imposed no limitations. There was a significant difference between the recommendations of specialists and clinics for physical rest, with 37% of clinics reporting no restriction and 54% of specialists reporting a restriction of 5–10 kg (*p* = 0.045).

### Hysterectomy

Among medical specialists, 61% recommended sick leave of 3–6 weeks, and 22% advised 6–8 weeks. Similarly, 61% of clinics recommended 3–6 weeks, while 12% suggested either shorter (1–3 weeks) or longer (6–8 weeks) durations. Regarding lifting restrictions, 28% of specialists advised 1–5 kg, 48% 5–10 kg, and 19% 10–15 kg. Clinics reported similar recommendations, with 27% suggesting 1–5 kg, 39% 5–10 kg, and 14% imposing no restriction. A follow-up consultation within 4–6 weeks was recommended by 61% of both specialists and clinics. Restrictions on swimming and full baths were suggested by 74% of specialists for 4–6 weeks and by 15% for 6–8 weeks. Clinics showed a broader range, with 20% recommending 1–4 weeks, 51% 4–6 weeks, and 14% 6–8 weeks. For tampon use, 57% of specialists recommended 4–6 weeks of restriction, and 22% advised 6–8 weeks. Clinics recommended shorter durations more often, with 14% suggesting 1–4 weeks, 37% 4–6 weeks, and 18% 6–8 weeks. Regarding sexual activity, 63% of specialists and 49% of clinics advised abstinence for 4–6 weeks, while 28% and 29%, respectively, suggested 6–8 weeks. Sports restrictions were advised by 61% of specialists for 4–6 weeks and by 26% for 6–8 weeks. Among clinics, 18% recommended 1–4 weeks, 43% 4–6 weeks, and 16% 6–8 weeks.

### Cesarean section

Half of the medical specialists recommended sick leave of 3–6 weeks, and 24% advised 6–8 weeks. Among clinics, 43% recommended 3–6 weeks, and 22% suggested 6–8 weeks. Regarding lifting restrictions, 30% of specialists recommended a limit of 1–5 kg, and 50% advised 5–10 kg. In clinics, 25% recommended 1–5 kg, 39% 5–10 kg, and 16% imposed no restriction. A postoperative consultation within 4–6 weeks was advised by 61% of specialists and 55% of clinics. Restrictions on swimming and bathing were recommended by 56% of specialists and 43% of clinics for 4–6 weeks, while 28% and 22%, respectively, advised 6–8 weeks. With regard to tampon use, 52% of specialists and 37% of clinics recommended restrictions for 4–6 weeks, and 30% and 29%, respectively, for 6–8 weeks. Notably, 10% of clinics imposed no restrictions. For sexual activity, 54% of specialists recommended abstinence for 4–6 weeks and 33% for 6–8 weeks. Clinic recommendations were similar, with 41% advising 4–6 weeks and 22% 6–8 weeks; 16% recommended 1–4 weeks. Sports restrictions were recommended for 4–6 weeks by 44% of specialists and 39% of clinics. Longer restrictions of 6–8 weeks were suggested by 41% of specialists and 16% of clinics. The only significant difference between the recommendations of specialists and clinics was with respect to abstinence from sport (clinics: 39% 4–6 weeks; specialists: 44% 4–6 weeks, *p* = 0.021).

It has been determined that the two groups’ recommendations are based on different principles. A statistically significant discrepancy is observed between clinics and medical specialists (*p* = 0.001). Amongst the 54 medical specialists, 4% assert that they formulate their recommendations based on the present study situation. Furthermore, 22% of respondents indicated that their recommendations were informed by expert opinion, while 33% drew upon their established clinical practices. The majority of respondents, 41%, reported that they base their recommendations on personal experience. In contrast to the medical specialists, 41% of the clinics surveyed make their recommendations based on the current study situation. In addition, 18% of clinics refer to expert opinion, while 8% are based on individual in-house opinion. The data indicate that 29% of the recommendations are informed by personal experience.

## Discussion

The findings of this study demonstrate considerable heterogeneity in postoperative behavioral recommendations for gynecological procedures. This variability is evident both between hospitals and private practitioners, as well as within these groups. It is noteworthy that for procedures such as LLETZ conization, TVT, and laparoscopy, hospitals tend to recommend shorter restrictions in comparison to private practitioners. While these variations are statistically significant, they are not clinically relevant due to their negligible magnitude. In contrast, recommendations for hysterectomy and cesarean section demonstrate a greater degree of consistency across both groups. While hospitals more frequently cite evidence-based approaches as the basis for their recommendations, private practitioners rely more heavily on personal experience; however, it should be noted that there are currently no major studies or evidence-based approaches regarding the recommendations in German-speaking countries. This may also influence the tendency to refer to evidence-based practice in institutional settings, particularly academic settings.

A similar study by Naidu et al. [4] in England surveyed 235 hospitals and 1557 general practitioners regarding postoperative behavioral recommendations for gynecological procedures. The study differentiated between diagnostic/operative hysteroscopy, diagnostic/operative laparoscopy, and abdominal or vaginal surgery. The findings of this study are consistent with those of the present study, in that a wide range of recommendations were identified. For instance, sick leave durations following operative hysteroscopy ranged from 1 to 42 days, with a median of 7 days, aligning with the current study’s findings where 75% of respondents recommended 1 week of sick leave; however, recommendations concerning the impact on physical activity following laparoscopy diverge. Naidu et al. [4] proposed a reduction in moderate physical activity for 7–14 days, whereas the present study advises a period of rest spanning 2–4 weeks (median: 38%).

Other methods for developing postoperative recommendations, such as the Delphi method employed by Noordegraf et al. [9], involve multiple rounds of consensus building among experts. In this particular instance, a group of 75 medical practitioners, comprising a mere 5 gynecologists, were tasked with formulating recommendations for 38 distinct activities following laparoscopic benign surgery or vaginal/abdominal hysterectomy. The findings of this study are largely aligned with those of the present study.

Bouwsma et al. [10] conducted a prospective study in the Netherlands, comparing recommended with actual durations of recovery activities in 304 patients following laparoscopic, vaginal, and abdominal hysterectomy or adnexal surgery. The findings revealed that in the majority of cases, the observed recovery times exceeded the recommended durations. This finding highlights a critical gap in the availability of evidence-based guidelines, underscoring the necessity for alternative approaches to inform clinical practice. To close this gap, an expert panel of gynecologists and general practitioners was convened to formulate recommendations, which were subsequently made accessible to patients via a preoperative website. The analysis revealed that no consistent factors could be identified, with the exception of length of sick leave. Specific recommendations for tampons, full baths, sexual activity, or follow-up consultations were absent, while everyday activities like stair climbing and walking were more precisely defined.

The Association of the Scientific Medical Societies in Germany (AWMF) S3 guidelines on cesarean section [11] provide a structured, evidence-based framework of postpartum and postnatal care recommendations for both mother and newborn. By aligning closely with established international standards (e.g., NICE guidelines) and advocating structured postoperative monitoring protocols, it offers a unified model of care that underscores the need for harmonization and standardization in post-cesarean management.

The WHO postnatal care recommendations [12] outline a structured approach to maternal and newborn care in the critical early postpartum period. The guidelines call for a minimum 24‑h facility-based observation with continuous monitoring, followed by timely follow-up contacts (e.g., early home visits) to ensure consistent care across settings. This harmonized approach underscores that standardized postnatal practices are vital to improving outcomes and addressing variability in care.

Recognizing the higher perioperative risks in obesity, the American College of Obstetricians and Gynecologists (ACOG) guidance [13] provides structured recommendations for the care of obese women undergoing gynecologic surgery. This comprehensive model spans preoperative risk counselling, evidence-driven surgical choices, and tailored postoperative management. It underscores that standardized protocols and consistent practices are essential to reduce variability and improve surgical outcomes in this high-risk population.

Existing guidelines are largely limited to those published by the Royal College of Obstetricians and Gynaecologists (RCOG) [14]. These guidelines cover operative hysteroscopy, diagnostic and operative laparoscopy, abdominal and vaginal surgery, offering recommendations on light and strenuous activity, sexual activity, sick leave, driving, and resuming normal activities. While there is some consistency with the present findings, specific timeframes are lacking for certain factors, so an individualized approach is recommended instead. For instance, the recommendation for resuming sexual activity following hysteroscopy is “once bleeding has ceased”, whereas the recommendation for light activity following laparoscopy is “as soon as possible”, and for sexual activity, “when the patient feels ready”. Furthermore, in terms of physical rest, the RCOG categorizes its recommendations into “low”, “moderate” and “high” activity. The results of our study indicate that clinics would probably not impose restrictions on “low” physical activity.

The study is subject to certain methodological limitations, which are particularly attributable to the utilization of a questionnaire with predefined response options. This may not fully reflect the spectrum of clinical practice. Furthermore, the geographical restriction of the surveyed medical specialists to Tyrol or the clinics in Austria may introduce a potential bias.

The underlying principle of the provided guidance is to minimize complications arising from gynecological operations; however, there is an absence of research addressing the consequences of patients disregarding these recommendations. To date, no study has been found that documents an increased incidence of complications in cases where these recommendations are not followed.

The study emphasizes the dearth of evidence-based recommendations at both the national and international levels, signifying that decisions are frequently made on the basis of experience or expert opinion, resulting in considerable variability. These results emphasize the necessity for the urgent development of standardized guidelines to optimize and standardize the postoperative care of gynecological patients. In future studies, the development and validation of such guidelines should be further investigated, and individual needs and comorbidities of patients should be considered in addition to the various aspects of postoperative care.
